# Supplementary material for: Metabolic remodeling by TIGAR overexpression is a therapeutic target in esophageal squamous-cell carcinoma
Source: Theranostics. 2020 Feb 10;10(8):3488–502. doi: 10.7150/thno.41427 (PMC7069087; doi:10.7150/thno.41427)
Supplement: Supplementary file 1 — Supplementary tables. [file thnov10p3488s1.pdf]

Table S1. Characteristics of 225 patients with esophageal squamous-cell carcinoma in this study

Sample ID	Age (year)	Sex*	Smoker[#]	Drinker[#]	TNM[§] stage	T stage	N stage	M stage	Survival status	Survival time (month)
150113	70	M	Yes	No	III	3	1	0	Deceased	Missing
150116	72	M	Yes	No	III	3	2	0	Deceased	12.4
150133	64	M	Yes	Yes	II	2	0	0	Alive	46.2
150146	52	M	Yes	Yes	II	3	0	0	Deceased	25.1
150152	61	M	Yes	Yes	III	3	3	0	Deceased	Missing
150159	63	M	Yes	No	II	2	0	0	Alive	45.8
150201	63	M	No	No	II	3	0	0	Alive	45.7
150203	61	M	Yes	No	I	3	0	0	Deceased	38.6
150208	60	M	Yes	Yes	III	2	2	0	Deceased	17.6
150219	68	M	No	No	III	3	3	0	Deceased	21.6
150228	61	M	Yes	Yes	I	1	0	0	Alive	45.4
150229	67	F	No	No	II	2	0	0	Alive	45.3
150233	53	M	Yes	Yes	III	3	1	0	Deceased	11.7
150234	61	M	Yes	No	II	2	0	0	Alive	44.8
150241	62	M	Yes	Yes	III	3	2	0	Deceased	10.0
150302	62	M	No	No	III	3	1	0	Deceased	20.8
150304	58	M	Yes	No	II	2	1	0	Deceased	23.9
150311	63	M	Yes	Yes	III	3	2	0	Deceased	Missing
150312	62	M	Yes	Yes	II	3	0	0	Alive	44.9
150313	69	M	Yes	No	II	3	0	0	Alive	44.9
150319	77	M	Yes	No	II	2	1	0	Deceased	3.4
150322	70	M	No	No	II	3	0	0	Deceased	32.9
150323	66	M	No	No	III	3	1	0	Deceased	31.9
150324	51	M	Yes	Yes	II	2	0	0	Alive	44.8
150326	60	M	Yes	Yes	II	3	0	0	Deceased	Missing
150331	69	M	Yes	Yes	II	2	1	0	Deceased	19.6
150333	69	M	Yes	No	II	3	0	0	Deceased	27.6
150334	56	M	Yes	No	IV	3	2	1	Deceased	30.8
150339	55	M	Yes	Yes	II	2	0	0	Alive	44.7
150347	73	M	No	No	II	2	0	0	Deceased	Missing
150348	74	M	Yes	Yes	I	1	0	0	Deceased	33.7
150355	58	M	Yes	Yes	III	3	1	0	Deceased	0.5
150374	57	F	No	No	II	2	1	0	Alive	44.4
150378	60	M	No	No	I	1	0	0	Alive	44.4
150385	71	F	No	No	III	3	2	0	Alive	44.3
150388	65	M	Yes	Yes	II	3	0	0	Deceased	7.9
150389	64	M	Yes	Yes	I	1	0	0	Deceased	Missing
150395	62	F	No	No	III	3	1	0	Deceased	11.9
150411	50	M	Yes	Yes	II	3	0	0	Alive	43.8
150414	65	M	Yes	Yes	II	2	0	0	Alive	43.8

150418	65	M	Yes	Yes	III	3	2	0	Deceased	6.6
150419	58	F	No	No	III	3	1	0	Alive	43.7
150423	62	M	Yes	Yes	II	3	0	0	Alive	43.7
150429	52	M	Yes	Yes	III	3	3	0	Deceased	7.3
150431	59	M	Yes	Yes	II	2	0	0	Alive	43.6
150432	57	F	No	No	II	3	0	0	Alive	43.5
150434	62	M	Yes	Yes	III	3	3	0	Deceased	Missing
150436	76	M	Yes	Yes	IV	3	2	1	Deceased	24.4
150441	50	M	Yes	Yes	II	2	1	0	Alive	42.5
150454	62	M	No	No	III	3	2	0	Alive	42.3
150455	65	M	No	Yes	III	4	1	0	Deceased	4.9
150458	68	F	No	No	II	3	0	0	Deceased	34.3
150459	59	F	No	No	II	3	0	0	Alive	42.2
150468	64	M	Yes	Yes	IV	3	2	1	Deceased	32.1
150472	74	M	No	No	III	3	1	0	Deceased	27.0
150473	58	M	No	Yes	I	3	0	0	Alive	42.1
150474	68	M	Yes	No	III	3	1	0	Alive	42.1
150476	60	M	No	No	II	2	0	0	Alive	42.0
150481	65	F	No	No	IV	3	2	1	Alive	41.8
150482	70	M	Yes	Yes	II	3	0	0	Deceased	2.5
150502	52	M	Yes	Yes	III	3	1	0	Deceased	7.5
150506	73	M	Yes	No	IV	3	2	1	Deceased	4.4
150510	55	M	Yes	Yes	II	3	0	0	Alive	41.8
150513	61	M	Yes	No	II	3	0	0	Deceased	3.5
150526	60	M	Yes	Yes	II	2	0	0	Alive	41.6
150527	67	F	No	No	II	2	1	0	Deceased	7.2
150528	68	M	Yes	Yes	II	3	0	0	Alive	41.6
150531	57	M	Yes	Yes	III	2	2	0	Alive	41.8
150541	69	M	Yes	Yes	I	1	0	0	Alive	41.7
150543	74	M	Yes	Yes	III	3	1	0	Deceased	14.1
150555	66	M	Yes	No	II	3	0	0	Deceased	23.7
150557	64	F	No	No	II	2	0	0	Alive	41.5
150560	70	M	Yes	Yes	II	3	0	0	Deceased	32.9
150561	58	M	Yes	No	III	3	1	0	Deceased	9.0
150582	48	F	No	No	I	1	0	0	Alive	41.2
150585	65	M	Yes	Yes	III	2	2	0	Deceased	1.6
150601	64	M	Yes	No	III	4	0	0	Alive	41.1
150603	78	M	Yes	Yes	III	4	1	0	Alive	41.1
150606	58	F	No	No	III	3	1	0	Deceased	10.6
150609	49	M	Yes	Yes	III	3	1	0	Deceased	12.6
150613	53	F	No	No	II	1	1	0	Alive	41.0
150614	67	M	Yes	Yes	III	3	1	0	Deceased	1.9
150617	74	F	No	No	II	2	0	0	Deceased	28.8

150618	52	M	Yes	Yes	II	3	0	0	Alive	40.9
150622	75	M	No	No	II	2	0	0	Deceased	21.5
150623	62	M	No	Yes	II	3	0	0	Deceased	6.3
150627	58	M	Yes	No	III	3	3	0	Deceased	26.6
150631	61	M	Yes	Yes	II	3	0	0	Alive	40.8
150641	52	M	Yes	Yes	III	3	1	0	Deceased	6.0
150645	71	F	No	No	II	2	0	0	Deceased	12.6
150654	61	M	Yes	Yes	III	3	1	0	Alive	40.5
150660	71	F	No	No	III	2	2	0	Deceased	15.7
150662	57	M	No	No	II	1	1	0	Alive	40.4
150664	58	M	Yes	Yes	III	2	2	0	Deceased	12.2
150666	48	M	Yes	Yes	I	1	0	0	Deceased	6.2
150667	56	M	Yes	Yes	II	1	1	0	Deceased	22.8
150671	65	F	No	No	II	2	0	0	Alive	40.2
150703	44	M	No	No	II	2	1	0	Alive	40.1
150705	63	M	Yes	No	I	2	0	0	Alive	40.1
150707	59	M	Yes	Yes	II	3	0	0	Alive	40.1
150710	67	F	No	No	II	2	0	0	Alive	40.0
150724	71	M	Yes	No	II	3	0	0	Alive	39.9
150726	69	M	Yes	Yes	II	2	0	0	Deceased	9.3
150730	65	M	Yes	No	I	1	0	0	Alive	39.8
150734	75	F	No	No	II	2	1	0	Alive	39.7
150735	59	M	Yes	No	III	2	2	0	Deceased	22.8
150736	60	M	Yes	No	III	3	1	0	Alive	39.7
150739	70	M	Yes	Yes	II	2	0	0	Deceased	7.9
150741	73	M	Yes	No	III	4	3	0	Deceased	21.4
150745	64	F	No	No	III	2	3	0	Alive	39.5
150747	64	M	Yes	Yes	II	3	0	0	Alive	39.5
150749	71	M	Yes	No	III	3	2	0	Alive	39.5
150758	53	M	Yes	No	III	3	2	0	Deceased	2.7
150762	59	M	Yes	Yes	III	3	1	0	Deceased	37.6
150770	73	F	No	No	II	3	0	0	Deceased	10.5
150801	63	M	Yes	Yes	III	3	1	0	Deceased	2.4
150805	51	F	No	No	II	3	0	0	Alive	39.0
150806	61	M	Yes	Yes	II	3	0	0	Alive	39.0
150807	64	M	No	Yes	III	1	3	0	Deceased	9.0
150812	52	M	Yes	Yes	III	3	1	0	Deceased	5.4
150813	50	M	No	No	I	2	0	0	Deceased	1.6
150843	58	M	Yes	Yes	III	3	1	0	Deceased	23.0
150845	66	F	No	No	I	1	0	0	Alive	38.3
150846	74	M	No	No	III	3	1	0	Alive	38.3
150848	53	F	No	No	III	3	1	0	Alive	38.3
150854	60	F	No	No	II	3	0	0	Deceased	28.0

150901	57	F	No	No	I	1	0	0	Alive	38.0
150903	51	M	Yes	Yes	III	3	1	0	Alive	38.1
150906	55	M	Yes	No	III	3	2	0	Alive	37.9
150909	72	M	Yes	No	II	2	0	0	Alive	37.9
150914	63	M	Yes	Yes	III	3	1	0	Deceased	5.3
150917	72	M	Yes	No	II	3	0	0	Deceased	Missing
150918	63	F	No	No	III	2	3	0	Alive	37.6
150923	74	M	Yes	No	II	3	0	0	Deceased	23.5
150933	51	M	Yes	Yes	II	3	0	0	Alive	37.4
150934	61	M	Yes	Yes	I	3	0	0	Alive	37.4
150935	61	F	No	No	III	3	1	0	Alive	37.4
150936	65	M	Yes	Yes	II	3	0	0	Alive	37.4
150947	61	M	No	Yes	II	3	0	0	Alive	37.5
150953	68	M	Yes	Yes	III	3	1	0	Deceased	5.8
150954	65	F	No	No	II	3	0	0	Alive	37.5
151002	58	F	No	No	II	3	0	0	Alive	37.2
151011	67	M	Yes	No	III	3	1	0	Deceased	4.3
151012	69	F	No	No	II	3	0	0	Deceased	32.7
151016	63	M	Yes	Yes	II	2	0	0	Deceased	34.3
151020	68	F	No	No	II	2	0	0	Alive	37.0
151021	62	M	Yes	Yes	II	3	0	0	Deceased	15.8
151024	55	F	No	No	II	3	0	0	Alive	36.9
151033	50	M	Yes	Yes	II	3	0	0	Alive	36.7
151040	67	M	No	No	II	2	1	0	Alive	36.7
151044	52	M	Yes	Yes	III	3	1	0	Deceased	25.2
151047	65	M	Yes	Yes	II	3	0	0	Alive	36.5
151049	52	M	Yes	Yes	III	3	1	0	Alive	36.5
151050	46	M	Yes	Yes	II	3	0	0	Alive	36.5
151052	61	M	Yes	Yes	II	2	1	0	Deceased	19.9
151053	56	F	No	No	I	1	0	0	Alive	36.5
151107	68	M	No	No	III	3	3	0	Alive	36.5
151116	74	M	Yes	Yes	III	3	1	0	Alive	36.4
151131	69	F	No	Yes	III	3	1	0	Deceased	0.6
151133	67	F	No	No	III	3	1	0	Alive	36.0
151138	49	M	No	No	II	3	0	0	Alive	36.0
151202	60	F	No	No	III	3	1	0	Deceased	Missing
151209	52	M	No	No	II	2	1	0	Deceased	8.4
151212	70	M	No	No	II	2	1	0	Deceased	14.5
151243	63	F	No	No	II	3	0	0	Alive	34.1
151256	60	M	Yes	Yes	III	3	1	0	Deceased	0.1
160103	61	M	Yes	Yes	I	1	0	0	Alive	33.7
160105	66	F	No	No	II	2	0	0	Alive	33.7
160106	56	F	No	No	III	3	1	0	Alive	33.7

160110	62	F	No	No	I	3	0	0	Alive	33.8
160111	66	F	No	Yes	II	3	0	0	Deceased	20.6
160114	72	F	No	No	II	3	0	0	Alive	33.8
160117	75	M	Yes	Yes	III	3	1	0	Deceased	14.4
160119	67	M	No	No	I	1	0	0	Alive	33.8
160131	57	M	Yes	No	III	3	2	0	Deceased	19.8
160139	65	M	Yes	Yes	I	1	0	0	Alive	33.5
160140	56	M	Yes	Yes	III	3	2	0	Deceased	23.4
160143	62	M	Yes	Yes	II	2	0	0	Alive	33.4
160151	53	M	Yes	Yes	II	3	0	0	Alive	33.3
160161	59	M	Yes	No	IV	3	2	1	Deceased	17.8
160204	64	M	Yes	No	II	2	1	0	Alive	32.6
160205	62	M	Yes	Yes	I	1	0	0	Alive	32.3
160209	57	M	Yes	No	II	2	1	0	Alive	32.6
160211	72	M	Yes	Yes	II	3	0	0	Alive	32.6
160218	64	M	Yes	No	I	1	0	0	Alive	32.5
160219	66	M	Yes	No	III	3	3	0	Deceased	20.1
160220	61	F	No	No	III	4	2	0	Deceased	24.9
160222	52	M	Yes	Yes	II	3	0	0	Alive	32.5
160227	72	F	No	No	I	1	0	0	Alive	32.5
160228	71	M	Yes	No	III	3	1	0	Alive	32.5
160236	62	M	Yes	Yes	III	3	3	0	Deceased	18.9
160238	63	M	No	Yes	III	3	2	0	Deceased	12.8
160246	70	F	No	No	II	3	0	0	Alive	32.3
160248	49	M	Yes	Yes	II	3	0	0	Alive	32.3
160301	67	F	No	No	III	3	1	0	Deceased	7.6
160303	55	M	Yes	Yes	III	3	1	0	Alive	32.3
160309	63	M	Yes	No	II	3	0	0	Alive	32.2
160310	69	M	Yes	Yes	II	3	0	0	Deceased	9.5
160311	52	M	Yes	Yes	III	3	1	0	Deceased	18.8
160319	57	M	Yes	Yes	III	3	2	0	Deceased	21.6
160320	64	M	Yes	Yes	II	3	0	0	Alive	32.2
160324	53	M	Yes	Yes	II	3	0	0	Deceased	16.4
160326	65	M	Yes	Yes	II	3	0	0	Alive	32.0
160333	61	M	Yes	Yes	III	2	3	0	Deceased	13.3
160334	64	M	Yes	No	II	3	0	0	Alive	31.9
160343	66	M	Yes	Yes	II	3	0	0	Alive	31.8
160372	53	F	No	No	I	1	0	0	Alive	31.4
160375	60	F	No	No	III	3	1	0	Alive	31.3
160413	57	M	No	Yes	III	3	1	0	Deceased	19.6
160414	62	M	No	No	I	1	0	0	Alive	31.2
160421	65	F	No	No	I	1	0	0	Alive	31.1
160430	71	M	No	No	III	3	2	0	Deceased	18.5

160432	73	M	Yes	No	III	3	3	0	Deceased	5.6
160439	66	M	Yes	Yes	II	3	0	0	Deceased	10.1
160441	75	M	No	No	II	3	0	0	Deceased	19.6
160443	61	F	No	No	III	3	3	0	Deceased	19.3
160453	72	M	Yes	Yes	II	2	0	0	Alive	31.6
160458	66	M	Yes	Yes	II	3	0	0	Deceased	22.4
160460	59	M	Yes	Yes	II	3	0	0	Alive	30.6
160461	63	M	Yes	Yes	II	2	1	0	Deceased	9.8
160466	66	M	Yes	Yes	III	3	1	0	Alive	30.5
160467	73	M	No	Yes	I	1	0	0	Alive	30.3
160469	58	F	No	No	I	1	0	0	Alive	30.6
160473	48	M	Yes	Yes	II	3	0	0	Alive	30.6
160474	51	M	yes	Yes	III	3	1	0	Alive	30.6

*M, male; F, female. #Individuals who smoked an average of <1 cigarette/d and for <1 year in their lifetime were defined as nonsmokers; otherwise, they were defined as smokers. Individuals were classified as drinkers if they drank at least twice a week and continuously for at least 1 year during their lifetime; otherwise, they were defined as nondrinkers. §Tumor TNM staging components, including tumor (T), lymph node (N) and metastasis (M), were reviewed by at least 3 pathologists and defined according to the American Joint Committee on Cancer (AJCC) 7th edition.

Table S2. Small interfering RNA sequences for expression knockdown of the investigated genes in this study

Gene symbol	Small interfering RNA sequence (5' → 3')
CCND1	#1: GUUCGUGGCCUCUAAGAUG
	#2: CCGAGAAGCUGUGCAUCUA
	#3: GAACAGAAGUGCGAGGAGG
CTTN	#1: GAACAAGACCGAAUGGAUA
	#2: GAAUAUCAGUCGAAACUUU
	#3: GGACAGAGUUGAUCAGUCU
PPFIA1	#1: GAAGAAAGGUUACGACAGA
	#2: GAACGACACUCGCCUAUGG
	#3: GUGAUUCGCUGGAUCCUGU
FADD	#1: CAGCAUUUAACGUCAUAUG
	#2: UGCAGCAUUUAACGUCAUA
	#3: GUGCAGCAUUUAACGUCAU
ANO1	#1: GCAGAGAGGCCGAGUUUCU
	#2: GCACGAUUGUCUAUGAGAU
	#3: UUACGUGGCGUUCUUCAAA
ORAOV1	#1: GAAAUUCCCUUAUGAUGAC
	#2: GCAGAAGGUUCCGGACUUU
	#3: GAAGAAGGCAGUAGUUUGG
ACTL6A	#1: GGAAAUAGAUGGCGAUAAA
	#2: GAACGGAGGUUUAGCUCAU
	#3: GAUGCCAACUGUUCAUUAU
FXR1	#1: CCAUACAGCUUACUUGAUA
	#2: GUAAACAUCUUAAGUGACA
	#3: CGUACGAAGUUGAUGCUUA
KCNMB3	#1: UGAAGAGGCUGUCCAGAUA
	#2: UCAGAAAGCUCUCCUACAU
	#3: GAGACAGACUACAGUGAUG
ZNF639	#1: GAGCAUGCAUGUAAAUUAA
	#2: CAACAAAGAUGAUGAUUCU
	#3: GGAGUAGACUUCACACAAA
MRPL47	#1: GAAACGAGCCCGCAUCAAA
	#2: GUCCAGAGCGGUUAGAUAA
	#3: GUUAUACCUUGGCACCUAA
NDUFS6	#1: GUUUGUAGGUCGUCAGAAA
	#2: GGUGGAGACUCGGGUGAUA
	#3: ACACUGGCCAGGUUUAUGA
MRPL36	#1: GGUGGUACGUCUACUGUAA
	#2: ACAAGACUGUCCUUAAGAA
	#3: GGUGAACCCUCUGCUCUAU
BRD9	#1: CAAUGAAGAUACAGCUGUU

	#2: GGCCAGAUACCGUGUACUA
	#3: CACGCAGGCUUUAAGAUGA
LPCAT1	#1: GAACUCUGAUCCAGUAUAU
	#2: GGACAGAUACUCAGAAAGA
	#3: GGAAAGUGGCCACAGAUAA
CLPTM1L	#1: GCACUUACAGCGAAUCUGA
	#2: GAAGAAAUCAACCUGCUCA
	#3: GAAACAAUGGGACGCUGUA
MCM7	#1: GGAAAUAUCCCUCGUAGUA
	#2: GGAGAGAACACAAGGAUUG
	#3: UGUCAUACAUUGAUCGACU
PMS2P1	#1: GGACUUAACUGGAGAGGUU
	#2: GAAAGGAGCUUCUGAUUGC
	#3: CAAUGUAGCUCUGGAUAUA
POLR2J	#1: UGCGGACACUGGAAAGAAA
	#2: CAGGAGACCAGGUGUCUGA
	#3: GAAGAAAACCCGAGGUGAU
TAF6	#1: CUACCGAGCUUGCAAGUUU
	#2: UGGCUGGAAUUUACUAUAU
	#3: GGUAAGACUUUGUCAAGGG
BUD31	#1: GAAAGUGGAAUCUCUGUGG
	#2: CUACAUCUUCGACCUCUUU
	#3: GCGGAAAGCCAUCAGCAGA
ATP5J2	#1: CUUCAGUCCUAGUGGCAUU
	#2: CAGUGAAGGACAAGAAACU
	#3: AAACAUGACUGGUACGUGU
POP7	#1: CCAGUGGAAUACACCCUUA
	#2: GAUCUACAUUCACGGCUUG
	#3: ACAUGAAGACGGACUUUAA
ARPC1A	#1: ACGAAGUGCACAUCUAUAA
	#2: GAAUUAAUCGCGCAGCUAC
	#3: GUGGCACGAUGGCGAGGAA
CPSF4	#1: UGAAUUACCUCGUGGGAUU
	#2: UUACAAGUGUGGCGAGAAA
	#3: GCGCUGCUGUCUGUGAAUU
PDAP1	#1: CAAAAUAAAUUCCCGUGAA
	#2: GCAAAAGCGCAAAGGCGUU
	#3: CUGAGGAGAUCGACGCGCA
BRI3	#1: UGGAGCCACCUUCGCUUAA
	#2: GCCUUGAGGAAGCGACGAU
	#3: GCUCAUGACAACUCAAUAA
PTCD1	#1: AACCAGAGGUGGAUACUAA
	#2: GAACUACACCUAUCUCAUC

	#3: GGAUGUGCCUCGAUGUGUU
PPP1R35	#1: GCACAAAGAAAGUGAUUUA
	#2: AAACUUCAGUCUCCGAUUA
	#3: GAAAUUGACAGACGAAUAU
LRWD1	#1: GCAUCGUGCUCCACAAGUA
	#2: GAAGUCGGCUGUCAGGGAU
	#3: GAUUGGAGCUGCUUUCCGA
C7orf59	#1: GAACACACACUGCUGGUGA
	#2: GCGCCUGUCUGUGGUCUUU
	#3: GUGCAGUGCUGGCGUCAUC
UFSP1	#1: AGAGGUGAGUGCAGCCUUU
	#2: GGUAUUGGACCCUCACUAC
	#3: CUGCCUCGCUCACUUCGGA
SPDYE3	#1: GAGAAAGGAUGGCAAAGAU
	#2: CAAACGGGCUGCAGAGAUG
	#3: GAAACGAGCUCUGUGGAGU
SEC61G	#1: GCACUAAACCUGAUAGAAA
	#2: GUAAAGGACUCCAUUCGGC
	#3: GAUUUGCUAUAAUGGGAUU
VOPP1	#1: GAUGAACCCUGUCGGGAAU
	#2: GAGCCAGCCUUCAAUGUGU
	#3: GACUCUAUCCAACCUAUUA
PSMA6	#1: GAGCAAGGCCCUCAGGUAU
	#2: CCUGACAAAUUAUUGGAUU
	#3: CAAGUAUGGCUAUGAGAUU
SRP54	#1: GAAGAGGUAUUGAAUGCUA
	#2: GAAGACCUGUUUAAUAUGU
	#3: GAAAUGAACAGGAGUCAAU
KIAA0391	#1: GGGAAUCACUGCAGGUUUA
	#2: AAACUAACCUUUCAGCGUA
	#3: GGGAUAAACUUAAGGAAGA
BAZ1A	#1: CAAGUUAGAUUGCCAGUUA
	#2: GGAAUUAGAUCAAGAUAUG
	#3: GGUAUGAGCUGUUAAGUGA
SNX6	#1: GAUGAAGACCUCAAACUUU
	#2: UAAAUCAGCAGAUGGAGUA
	#3: CAAGAAGAGUUGCUGCAUU
BRMS1L	#1: UUAAUAGGCUCGUGGCUGA
	#2: GGCACGAGCUGGCGUUUGU
	#3: UGGCUGAGGUGGUCCGUAU
SPTSSA	#1: GCACUACUUUGAAAUCGUA
	#2: GAGCGGACGGUGUUCAAUU
	#3: AAGCAGAUGUCCUGGUUCU

TIGAR	#1: GGAAGGAAGAGAAGUUAAA
	#2: CCUACAGGAUCAUCUAAAU
	#3: GCUGGUAUAUUUCUGAAUA
METTL4	#1: GCAAUGAAGUUCUCAAAUU
	#2: CUACGAAGGUCUUAUACUG
	#3: GUUGUCAGCUGGGUGGUUA
RTEL1	#1: GACCAUCAGUGCUUACUAU
	#2: GACAUUAUCCAGAUUGUGU
	#3: UAUUCAUGCCGUACAAUUA
YTHDF1	#1: GAACAUGCCAGUUUCAAAG
	#2: GGACAGUCAAAUCAGAGUA
	#3: CGACAUCCACCGCUCCAUU
ARFGAP1	#1: ACAUUGAGCUUGAGAAGAU
	#2: GGGAUAAGGUGGUCGCUCU
	#3: GGGAGUCGGUAGUAAGGGA
ZGPAT	#1: GAAGAUGACUGAGUUCUAG
	#2: CUACUACACAGUCAAGUUU
	#3: UCAGAGACCGUUCCUAAAG
ABHD16B	#1: GCAACGAGAUCGACACUAU
	#2: GAGAUGGGCUGUCUGUCUG
	#3: GCACUUCAACCUCAACGUG
POLB	#1: GAAUUGGGCUGAAAUAUUU
	#2: CAAGGAAGUUUGUAGAUGA
	#3: GAUACGAGUUCAUCCAUCA
AP3M2	#1: GGACAGUAGUUCCCUUGGA
	#2: CAAUGUAGUUGUGGUUUAU
	#3: GUUCAGAGCCAGUGAUCAA
CRKL	#1: CCGAAGACCUGCCCUUUAA
	#2: GAAGAUAACCUGGAAUAUG
	#3: AAUAGGAAUUCCAACAGUU
SMARCB1	#1: GAAACUACCUCCGUAUGUU
	#2: CCACAACCAUCAACAGGAA
	#3: GUGACGAUCUGGAUUUGAA
SNRPD3	#1: GAAGAACGCACCCAUGUUA
	#2: GAACACCGGUGAGGUAUAU
	#3: CGAUUAAAGUACUGCAUGA
UFD1L	#1: AAUCAAGCCUGGAGAUAUU
	#2: GACCAAACCCGACAAGGCA
	#3: GAGCGUCAACCUUCAAGUG
ZNF74	#1: GAGCAAACCUCGAAACUAA
	#2: GAACCACUGUCUCAUUAAA
	#3: UGAAGGCGGUGCCCUCUCA
TRMT2A	#1: GAGAGAAGGCGCACCAUGA

	#2: AGAGAUCAGUACAGUGUGA
	#3: GAUAUUCCAGCCAGGGUAA
MED15	#1: CCAAGACCCGGGACGAAUA
	#2: CGACAAGAACGAAGACAGA
	#3: CGUCAGUGAUCCUAUGAAU
MGC16703	#1: GGACAAUGCUUUAAGUUUU
	#2: CAAUAACUCUACUCAUUUG
	#3: UCACAUCUAUAUCCAAGUU
MAD1L1	#1: GCUCUGGACUGGAUAUUUC
	#2: AGAGGGAGCUUGCCUUGAA
	#3: GAGCAGAUCCGUUCGAAGU
INTS1	#1: GGACAUCUAUUGCCUUUAA
	#2: GACCACAGAUCGAUACGUA
	#3: GGAAAUUACCGUUUACAAA
YEATS4	#1: GACAACAUCUCGUCAGCUA
	#2: CAACAGCAAUGAUGCAACA
	#3: UUACUAAACCUCCAUAUGA
CCT2	#1: GAAAUUGCCUCUACCUUUG
	#2: AAAGUUAGCUGUAGAAGCA
	#3: GGAGGAAGUUUGGCAGAUU
CPSF6	#1: GAAGAAAGCUUUGACAUCA
	#2: GAAGAAGAAUGUACUACUA
	#3: GGAGAUAUGUCUUUGUUAC
NUP107	#1: GGAAAUCUCUCCAUGGUUA
	#2: GAAAGUGUAUUCGCAGUUA
	#3: CAUCAGAGCUUAUUUGGAA
RAB3IP	#1: GGAUAAAGGGGUUGGGUAA
	#2: GAACAUCGGUGGUGUAUUA
	#3: GGAAAAUGUAAGACGUAAA
PARP1	#1: GAAAGUGUGUUCAACUAAU
	#2: GCAACAAACUGGAACAGAU
	#3: GAAGUCAUCGAUAUCUUUA
ADSS	#1: GAAACGAACCAGACCAAUA
	#2: AAACGGAGGUCGUAUAAUG
	#3: GGACAUCUCUAACAAACGG
NVL	#1: GAAACGAACCAGACCAAUA
	#2: AAACGGAGGUCGUAUAAUG
	#3: GGACAUCUCUAACAAACGG
TARBP1	#1: UCAGGGAUGUUAUUCAUUG
	#2: UAAGAGAGAAGACCAUUAU
	#3: CAGAGAAUGUAUUGCGGAU
ZNF124	#1: AAACUCAUAUUGCACAGAA
	#2: CCAGUUCCCUUCAGAAACA

	#3: CCAAUUGCCUUCAUUACCA
MAPKAPK2	#1: UCAAGGAACCAGAGAAUUA
	#2: GUAUUACGGCUAUGUCAAA
	#3: GUGGUAAUGAUCCAAAGUA
CEP170	#1: GAAGUAAAGUAACGAAAUC
	#2: CGUAACAUCUCUCGGAUUG
	#3: GAUUAUAAUAGGCCUGUUA
COG2	#1: GGACGGGCCUCGGGAGCUA
	#2: UGGAGGAAGUGGCUUGCAA
	#3: GGAGCCUGGUGUUGGUGAA
ZNF281	#1: GCACCACCGUGACGUAUUA
	#2: CCAAUGACCUUUAUCACUA
	#3: GCAGAAAGAAUACAGAUAA
AHCTF1	#1: GGGAGAAACUGCAGUACUA
	#2: GGAUAGCGACCUGGAAAUA
	#3: GGCGAAGGCAUCUCAGUUA
FLVCR1	#1: CGAAGACACAACAUAAAUA
	#2: CCUGAAGAGUACUCCUAUA
	#3: GGAUUGGGCUAACGCUAGU
PYCR2	#1: CCUGUCGGCUCACAAGAUA
	#2: GCAACAAGGAGACGGUGAA
	#3: GUCCAUGGCCGACCAAGAA
PPPDE1	#1: GCGCCACACUAAACUAUAA
	#2: GAAAGAGAUUCCUCGCUGG
	#3: AGAGUUGCCUCCCGAAGGA
IPO9	#1: UAUAAGAUCUUCACCAUGG
	#2: GACAAACGGCUACAGGAUA
	#3: CCUAAUGGGUUGAGAGAAU
SMYD3	#1: GGACAAACACGGACAGUAU
	#2: GUACUAAGGUCUACAUCGA
	#3: GGAGAGAAGCAGAAUAUUC
TTC13	#1: GCUAUGCUAUACAAAGGUU
	#2: AAUGAAGUGUGCCAGUAUA
	#3: CACUAAAGCUAUCCAACUG
ZNF669	#1: GUAAAGAAGAUGGUCAGUA
	#2: GAUACAAACCAUAUGGAGA
	#3: AAUCUUGAUCUGAACGAGA
NTPCR	#1: GCAUUGGGCUCAUGUUCUA
	#2: GCAGUACCCUGAAGGACAA
	#3: GACAUCCGCUUGACUCCUA
ZNF670	#1: UAAAGAAGGCAGUCAAUAU
	#2: GAAAGAGCUUAUAUGUGGU
	#3: UGUUGGAUCCCUAGACUAA

SNAP47	#1: GAAAGAAGGGAUACUGAUA
	#2: GUUAGAAGAUGCAUUGGUG
	#3: UAACAUCGCUGUCGCUCAG
C1orf96	#1: CCAAGUGCCUUAUUUGCUA
	#2: GCGAGUACAUGAAGCGCUA
	#3: AAACAGACCGAUACAGGAA
MRPL55	#1: GACAGAGUAUGCUCGCUAU
	#2: UCAAGCACCUCCCUGAGAA
	#3: GGACACUACUCUUACCGCA
SYT14	#1: UCACAGACAUCCCAACAUA
	#2: GCAAACAGACCACCCAAUA
	#3: CGGCAGAGUAUGAUGGAUA
JAG2	#1: GAACGGCGCUCGCUGCUAU
	#2: GCAAGGAAGCUGUGUGUAA
	#3: CCGGCCACCUGGACAAUAA
TRAF3	#1: GAUAAGGUGUUUAAGGAUA
	#2: GACAUCUGCUGGUGCAUUU
	#3: GGCCCAAACUGUUCUAGAA
XRCC3	#1: GGACCUGAAUCCCAGAAUU
	#2: GCUGAGAACGGCCUCCUUA
	#3: GGACCAGACUUGAAGAGAC
CCNK	#1: CAAAGCAACUCAAAGGUGA
	#2: CAAGUUUGAUUUACAGGUA
	#3: GAUCAUAGCAGUAGCAGUG
MTA1	#1: CCAUAGUGUUUGUCAUCUA
	#2: GCCUGGAGGCGCACAAAUA
	#3: GCGCACAAAUACCGGAACA
SNHG10	#1: GAGCAUGACUUCGUUAUUA
	#2: GGAUCGGCCAGCUGAAUUA
	#3: GGGAGUCGGUGAAGUACUU
TOR1A	#1: CAUCUUAAAUGCCGUGUUU
	#2: AGAAAGCCAUGUUCAUAUU
	#3: CUUCAAACAUCACCUUGUA
FPGS	#1: GACCAAGGAUGGCAGCUGU
	#2: GAAUGUAUCCUCCGAAGCU
	#3: GAACAUCAUCCACGUCACU
GLE1	#1: GGAAGGAGCUAAGUGCAAU
	#2: GGAACUACUUCUCCCUGUA
	#3: GCUCGGAUGUGCAGAACAA
ODF2	#1: GAAGGGAGACCGAGACAAA
	#2: CAAAUGACCUGCACGGACA
	#3: GCGGUUAAGUGACCUCUCU
PSMB7	#1: GGAAGAAACAGUCCAAACA

	#2: CAUCGCAGCUGGCAUCUUC
	#3: CCGCAGGAAUGCCGUCUUG
SET	#1: GAGGAAGGAUUAGAAGAUA
	#2: GGAUGAAGGUGAAGAAGAU
	#3: GAAGUCCACCGAAAUCAAA
SNAPC4	#1: GGACUUGGAUCCUGCCGAU
	#2: AAGGGUCGGUGGAAUUUAA
	#3: AAACGUGCCGGCUCAAGAA
SURF4	#1: GCAGGAACUUCGUGCAGUA
	#2: GGACUUCGCCGACCAGUUC
	#3: GCUCUUUGCCAUCAACGUA
VAV2	#1: UCACAGAGGCCAAGAAAUU
	#2: GAAAGUCUGCCACGAUAAA
	#3: GGGACGACAUCUACGAGGA
GTF3C5	#1: GCAGAUGUUCUACCAGUUA
	#2: GCAAGCAUACGUCAAUGUA
	#3: CGAAUCCGUUGUGGAAUGA
MED27	#1: GUAAAGGGAUAUAACGAGA
	#2: UGAAAGUGAUCGUCGUCAU
	#3: UGAAGGAUGGGAUGCGGAA
RABEPK	#1: CCACAGCUGUUCAUAUUUA
	#2: GGAUUCAGCUGACAAAGUA
	#3: GAAACCAGCUAUAUGUCUU
SDCCAG3	#1: GAACCUCGGCCUCUCGAAA
	#2: GAGCGGAAGUUAGAAGCAA
	#3: CCUCGUGGGCGUUGAGUGA
WDR5	#1: GAGAGUGGCUGGCAAGUUC
	#2: GACGAAAGCGUGAGGAUAU
	#3: CAGAGGAUAACCUUGUUUA
MAN1B1	#1: CUGCGAAGGUGGAGAGUUA
	#2: GAAAGAGUCAUCAGAAGUG
	#3: GGGAGUCGCUGCAAGAAUC
DOLK	#1: GAGAUCCGCUGGCCUGGAA
	#2: CUACAGUUAUGCUUGGAUU
	#3: CGCAAACAGUGGCCUAUUG
EXOSC2	#1: GAACGUAUAUGGGAGAAGA
	#2: GAAACACGCCAGAGGCUUU
	#3: GGGAUACAAUCACUACGGA
COBRA1	#1: GCCCUGAGGUGUUUCUGAA
	#2: GCAGUUCAGUGUCAUCGUC
	#3: UGACGCGACUGAUCUCAUG
NELF	#1: GAACGUCUACCACAAGGGA
	#2: GUACAGCGUUGACCGUGUG

	#3: CACCAAAGGUCAUGCUCAU
TOR1B	#1: GCAAUAAUGUAGACUGAUA
	#2: UGACAACCCUCGAAGAUAU
	#3: GGACUAAAGUAUUCCACAA
METTL11A	#1: GCAAGAGGGUGAGGAACUA
	#2: GGAAGUUUCUGCAGAGGUU
	#3: GCCAAGACCUACUGGAAAC
MRPS2	#1: CAUGAGCCAUUCCCUGUGA
	#2: CGGCAGUUCUCGUACCUGA
	#3: CCACAUGGCCUACCGCAAG
C9orf114	#1: GGAAGGAUCUCAAGCUGAU
	#2: GCAGGACCCUCGCACCAAA
	#3: CUACUUGGCCGGUCAGAUU
REXO4	#1: GGAAGUGCGUUUAUGACAA
	#2: GCGGAGCACUGUUCAAUUC
	#3: GAGGGGACAUCGAGCAUAA
DDX31	#1: UCGGUUAGCACAAGUGAUA
	#2: GGAGAGACAGUGCAUUAAG
	#3: CGCCGGAUCUCGCUUCUCA
C9orf69	#1: ACAACAACUUCGACGUGUA
	#2: GGUCUUCGUGGACAUGUGA
	#3: UCAAGACGCGCUGGCUGUA
UAP1L1	#1: UGAAGAAGGUCCCGUAUGU
	#2: CCAACGUGGUCAUGUUUGA
	#3: GAGAGGGUUUAGAAGUGUA
WDR85	#1: CAGGGUACCCGGCAAAUUU
	#2: GCAGAGAGGAUAACGAUGG
	#3: GAAGGGUGCAAGCGAGUUG
ZMYND19	#1: GAAUGGAAGUGGAUGCAGA
	#2: CUACAGACCCUAUAGAAGA
	#3: CUAAAUGUGACCCGGUAUU
PTRH1	#1: GGAGUCCGUUCCUGCAUUA
	#2: GCCAUGAGCCGAUGUGUUU
	#3: GACCUGAUCUUGGACCACA
QSOX2	#1: GACCUGAUCCCGUAUGAAA
	#2: GAUGCGGAUUUCUGGAAUA
	#3: UAAAGUGGGUUGGAUGUCA
C9orf142	#1: GGACAGAGCAUCCCUGACG
	#2: CAGGAGAGUCGCUCAUCAA
	#3: GAGUGCGGCUGAGGACAUC
DNLZ	#1: CGGAAGCGGGUGAGGAUGA
	#2: CUGCAAGGUCUGCGGGACU
	#3: GAAGAGAAAUAUCGAAGAG

LOC100272217	#1: CAGACUACUUGCUACAUUA
	#2: CAAGUACUAUCGCCAGACA
	#3: AAACUACCCACGAGCUUAA
SSB	#1: GAAAUGAAAUCUCUAGAAG
	#2: GAACAUUGCAUAAAGCAUU
	#3: AGAUAAAGGUCAAGUACUA
HAT1	#1: GCACAAACACGAAUGAUUU
	#2: GAAGAUUACCGGCGUGUUA
	#3: GCUACAGACUGGAUAUUAA
OLA1	#1: CAACAAGGCAGAAAUUAUA
	#2: UAACACACCUCAACAACCG
	#3: AGAAGUAUCUGGAAGCGAA
ABCF1	#1: GAUGAUGUCUGCACUGAUA
	#2: GGACAGAAACCACUCUUUA
	#3: CCAAAUCGAUGGUGACUUU
GTF2H4	#1: GAAACGGAGUCUAAGCUUU
	#2: GCUCUGAAGUGCAUCAAGA
	#3: GAACAGAAUGGCAUCAUGU
NEU1	#1: GAAAGAGACAGUCCAGCUA
	#2: CGGAAUCUCUCCCUGGAUA
	#3: CGAUGGAGCUUCAGCAAUG
SKIV2L	#1: GCUACAAGAACGUGAAGAA
	#2: GACCUCACCUGGUACGCCA
	#3: GGGAGAACUACCUGGGCAA
RDBP	#1: GAAGAUAUCCGGUGCAAAU
	#2: GAAUGAGGAUGCUCGCUCU
	#3: GAAAGCCGGAUGCAUAUAC
DHX16	#1: GGCAGGAGCUCAAAUAUAA
	#2: GAGAGUACCUGGCUAAGCG
	#3: GAACUGUCCUCCGCUACAU
MDC1	#1: GGACCAAACUUAACCAAGA
	#2: GAAGACAACUAUGGUGAUU
	#3: GAGCCCACAUCUCAGGUUA
EHMT2	#1: GAAGAGAGAUCAGCAUUAA
	#2: GAACAAGGACCUAAACAAA
	#3: GCAUAAAGUUCAACAGUAU
NRM	#1: CUUCAGAGGUCACUGUAUG
	#2: CCAAAGGCCCUGUGUUGUG
	#3: GCAUGGACAUCCCGGUACU
MRPS18B	#1: CAAGGAACCUGGUGUUGAA
	#2: GGAAUGGAAAGUAGGAUUA
	#3: CAAAUCUUCUUCUGAACUA
CCHCR1	#1: GAGACAACCUUGACAGAUG

	#2: UUAAGCAGCUGAAGGGACA
	#3: CCUGAUUGCUCGAAAGCUU
PRR3	#1: GGAGAGAGACGACCGUGUA
	#2: GAGAGAAGGCCACUGAUGA
	#3: GUACCCACCUGAGCUAUUC
ZNF146	#1: GCAAAUCCAACCUUACUGA
	#2: AGAAGUACCUCAUAAAACA
	#3: GCUCAGGAAAUACGCACUA
UBA2	#1: GCCGUUAGCCUGAUCAUCU
	#2: AGCAUGUCAUCCACAAGUA
	#3: CCUAUGUGAUGCUGAAGAC
HAUS5	#1: GAGGAAAGCCAAAGUAGAU
	#2: UCAAGGCCCUGCACGAUCA
	#3: GAAACCUACUCUGGUAUGG
ALKBH6	#1: ACAAGAACCUCCUGGUGUC
	#2: CGUCCAACAUCUCAAACUU
	#3: GCACCAGCACCUAGCGUAC
RHPN2	#1: GAGGAGGCAUUUACGAUUC
	#2: GCUUAGCCAUUGAUGAUGA
	#3: CGGAGUAAAUUGCAGAAUC
GLS	#1: CCCUGAAGCAGUUCGAAAU
	#2: CCAGGUUGAAAGAGUGUAU
	#3: GAGGCAUUCUACUGGAGAU
PRKAA1	#1: GAGGAGAGCUAUUUGAUUA
	#2: GCGUGUACGAAGGAAGAAU
	#3: CGGGAUCAGUUAGCAACUA

Table S3. Primers used for plasmid construction or quantitative real-time PCR (qRT-PCR) analysis

For qRT-PCR	Primer sequence (5' → 3')
TIGAR-F	GGGAAGAGTGCCCTGTGTTT
TIGAR-R	AATCCTGGAATACCGCTGTC
SLC1A5-F	TCCTCTTCACCCGCAAAAACCC
SLC1A5-R	CCACGCCATTATTCTCCTCCAC
GLS-F	CAGAAGGCACAGACATGGTTGG
GLS-R	GGCAGAAACCACCATTAGCCAG
GLUD1-F	CTCCAGACATGAGCACAGGTGA
GLUD1-R	CCAGTAGCAGAGATGCGTCCAT
ACTIN-F	CATTGCTGACAGGATGCAGAAGG
ACTIN-R	TGCTGGAAGGTGGACAGTGAGG
For lentiviral construction	Primer sequence (5' → 3')
pLVX–*TIGAR*-F	CCGGAATTCGCCACCATGGCTCGCTTCGCTCTG
pLVX–*TIGAR*-R	CGCGGATCCTTAGCGAGTTTCAGTCAGTCCAT
For expression plasmid construction	Primer sequence (5' → 3')
pcDNA3.1–*TIGAR*-F	CCCAAGCTTGCCACCATGGCTCGCTTCGCTCTG
pcDNA3.1–*TIGAR*-R	CCGCTCGAGTTAGCGAGTTTCAGTCAGTCCAT
For sgRNA vector construction	Primer sequence (5' → 3')
sgRNA1-F	CCGGCTTTCTCCAGTGATCTCATG
sgRNA1-R	AAACCATGAGATCACTGGAGAAAG
sgRNA2-F	CCGGTTAAATCCAGTTTCTGAAAG
sgRNA2-R	AAACCTTTCAGAAACTGGATTTAA
For *Tigar* KO mice identification	Primer sequence (5' → 3')
Tigar-GF1	AGCTTCCTCCACAGTGCTGAGATC
Tigar-GR1	GACCCTTAGCACCCAGAACTGAC
Tigar-GF2	GACATTTACAGGCTCGCATTAGCAC
Tigar-GR2	GTCAGGGTATGTGCATTTGACTG

Table S4. Score of immunohistochemical staining of TIGAR, phosphorylated-AMPK and glutaminase in esophageal squamous-cell carcinoma specimens

Sample ID	TIGAR			Phosphorylated-AMPK			Glutaminase		
	Positive	Intensity	IRS*	Positive	Intensity	IRS*	Positive	Intensity	IRS*
160408	2	2	4	2	1	2	2	2	4
160425	2	2	4	2	2	4	2	1	2
160442	2	3	6	2	2	4	2	3	6
160456	3	2	6	2	2	4	2	3	6
160466	3	3	9	2	3	6	4	3	12
160474	2	2	4	2	2	4	3	3	9
160475	4	2	8	3	3	9	3	2	6
160467	3	3	9	2	2	4	3	3	9
160458	3	3	9	3	3	9	4	3	12
160443	3	2	6	2	2	4	3	3	9
160430	2	3	6	2	1	2	2	2	4
160413	0	0	0	2	1	2	2	1	2
160432	3	2	6	2	2	4	2	2	4
160460	2	2	4	2	2	4	2	3	6
160468	2	1	2	2	2	4	2	3	6
160469	2	3	6	2	2	4	2	2	4
160461	3	1	3	2	1	2	2	1	2
160453	2	3	6	2	2	4	3	3	9
160414	2	1	2	2	1	2	2	2	4
160421	2	2	4	2	1	2	2	2	4
160439	2	3	6	2	3	6	2	3	6
160454	2	2	4	2	1	2	2	2	4
160470	3	3	9	2	2	4	2	3	6
160473	2	2	4	2	3	6	2	3	6
160463	3	1	3	2	3	6	4	3	12
160455	2	2	4	2	2	4	2	2	4
160441	2	3	6	2	3	6	3	3	9
160422	4	3	12	3	3	9	4	3	12

*IRS, immune reactive score. IRS was obtained by multiplying the score of positive and that of intensity. The labeling score of positive was defined as 1 (≤ 10%), 2 (11%–50%), 3 (51%–80%) and 4 (> 80%); the labeling score of intensity was estimated as negative (0), weak (1), moderate (2) and strong (3).
